# Chemiluminescence method for evaluating photooxidative degradation of dispensed drugs: a potential new drug information tool

**DOI:** 10.1186/s40780-024-00365-7

**Published:** 2024-07-24

**Authors:** Yuriko Murai, Kasumi Kudo, Hiroyuki Suzuki, Taisuke Konno, Yasuyuki Agatsuma, Hitoshi Nakamura

**Affiliations:** 1 Division of Clinical Pharmaceutics, Faculty of Pharmaceutical Sciences, Tohoku Medical and Pharmaceutical University, 4-4-1 Komatsushima, Aoba-Ku, Sendai, Miyagi 981-8558 Japan; 2 Division of Pharmaceutics, Faculty of Pharmaceutical Sciences, Tohoku Medical and Pharmaceutical University, 4-4-1 Komatsushima, Aoba-Ku, Sendai, Miyagi 981-8558 Japan; 3 Department of Pharmacy, Tohoku Medical and Pharmaceutical University Hospital, 1-12-1 Fukumuro, Miyagino-Ku, Sendai, Miyagi 983-8512 Japan

**Keywords:** Stability, Evaluation method, Oxidation, Visualization, Generic drug, Light, One-dose package, Immediate-release tablet

## Abstract

**Background:**

Dispensed drugs stored by patients are often in single-dose packages (SDPs) or are crushed and mixed after being removed from a press-through package (PTP) sheet. Information on their stability is extremely limited. To address this, we explored using chemiluminescence (CL) measurements to detect oxidative degradation.

**Methods:**

Eight amlodipine, 14 telmisartan, and two warfarin preparations were used as specimens. These preparations were stored at room temperature under various conditions, after which CL was measured. Cellopoly packaging paper was used for SDP. Three light conditions were used (Condition A: darkness, Condition B: indoor diffused light (approximately 400 lx), and Condition C: exposure to 4,000 lx). CL cumulative light output was measured every minute under nitrogen gas conduction and with a sample chamber temperature of 150 °C, for a maximum of 10 min. Luminescence images were obtained simultaneously with the CL measurements.

**Results:**

CL was observed on light-exposed tablet surfaces. For each preparation, an increase in the CL value was observed with the duration of light exposure. In the same preparation with the same exposure time, CL tended to be higher in the order of Condition A < B < C. Moreover, CL increased even when no changes in color were observed by the naked eye. A comparison between preparations with the same main ingredients showed differences in the rate of increase in CL with exposure, and each was found to show a different reactivity to light.

**Conclusions:**

To the best of our knowledge, this is the first study to visually capture the surface oxidation of tablets exposed to light using the CL method. The CL values, thought to be derived from photooxidation, increased with exposure of tablets and powders to light after SDP. This method can sensitively assess drug degradation due to photooxidation. Further research is needed to establish a CL method for assessing the stability of preparations in clinical settings.

**Supplementary Information:**

The online version contains supplementary material available at 10.1186/s40780-024-00365-7.

## Background

The drugs dispensed to patients must retain their quality until use. In this process, from provision by the manufacturer until consumption, drugs are removed from paper box or pillow packaging according to their prescription for individual patients. It is common for them to be removed from a press-through package (PTP) sheet and placed in single-dose packages (SDPs) with other drugs or ground and packaged, and then administered to patients. Societal factors, such as aging populations and strong patient need, drive this “individualization” of the dosage forms. As society ages, preparations such as SDPs or the crushing of solid medicines are needed because of decreases in the cognitive and motor functions of the hands and fingers, swallowing, and other functions. However, there is concern regarding the degradation due to the oxidation of drugs dispensed in these forms.

Much information on the physicochemical stability of drugs is provided by drug companies for individual drugs in a packaged state. Dispensed drugs are exposed to the environment (e.g., oxygen, temperature, humidity, and light), which is thought to significantly affect their stability [1–4]. Numerous studies have explored this topic [5–12]; but there are no limits to the “individualization” of preparations and post-preparation environments. Quality control is crucial when dispensing SDPs. Light exposure, especially, causes oxidation reactions in pharmaceuticals stored in the atmosphere, leading to changes such as coloration and decomposition. These changes are known to decrease potency or alter drug efficacy [13, 14]. Determining a method to quickly and easily assess the stability of drugs in forms in which they are maintained by patients under various conditions is a serious issue in clinical pharmacology.

Chemiluminescence (CL) occurs when molecules in a chemical reaction return from an excited state to the ground state [15]. As substance gradually oxidizes, oxides accumulate on its surface. During the oxidation process, intermediate peroxide radicals emit ultra-weak CL when they return to their ground state, which cannot be detected by the naked eye; however, the CL method can detect this light. Using a chemiluminescence analyzer (CLA) with a high-performance photomultiplier, luminescence from matter can be captured at the photon level** (**50 photons/cm^2^/sec, 10^–14^ W level) [16]. No light sources were used. A schematic of the CLA instrument was previously published elsewhere [17]. It is possible to capture oxidation in the very early stages before the occurrence of changes, such as discoloration and breakage,which can be seen by naked-eye examination. Oxidative degradation that cannot be detected with conventional methods can be detected by CL methods with high sensitivity. The CL measurement procedures are very simple: 1) the gas type in the sample chamber (nitrogen or oxygen), gas flow rate, and measurement temperature were set; 2) after reaching the set temperature, the sample is inserted; and 3) measurement is started. Therefore, CL methods are used in a wide range of industrial fields, including automobiles, food, bio-products, and medicine, for purposes such as shortening the period for testing the oxidative stability of materials in a mixed ingredient system, conducting inspections in the manufacturing process, before shipping, and during delivery, and elucidating the degradation mechanisms of various products [17, 18]. For example, in polymer chemistry, the Japanese Industrial Standards (JIS K 7351:2018) and International Organization for Standardization (ISO 4765:2022) have been published and CL is applied to assess the oxidation degradation of plastics. However, the only examples of applying CL to stability assessments of drug products were reported by Mizuno et al. [19, 20], and a series of studies conducted around 1990 by Mizugaki et al. [21–24]. Since then, little progress has been made, but improvements in the sample chambers of CL-measuring devices, and the integration of high-sensitivity CCD cameras now allow for visual data analysis of luminescence intensity.

The purpose of this study, based on societal needs and the evolution of measuring equipment, was to determine whether the CL method could be applied to compare and assess the stability of drugs after they have been dispensed.

## Methods

### Materials

The drugs used in the study are detailed in Table 1 (and Supplementary Table S1), comprising 8 preparations of amlodipine, 14 preparations of telmisartan, and 2 preparations of warfarin.

**Table 1:** Sample pharmaceuticals

	Main ingredient	Brand Name	Manufacturer	Lot	Color	Dosage form
#A-1	Amlodipine	Amlodipine Tablets 5 mg [MEIJI]	MEIJI SEIKA, Tokyo Japan	00938	white	film-coated tablet
#A-2		Amulodin® Tablets 5 mg	DAINIPPON SUMITOMO, Osaka, Japan	4016C	white	film-coated tablet
#A-3		Norvasc® Tablets 5 mg	PFIZER, Tokyo, Japan	ET1819	white	film-coated tablet
#A-4		Amlodipine Tablets 5 mg [SANDOZ]	SANDOZ, Tokyo, Japan	P0036	white	uncoated tablet
#A-5		Amlodipine OD Tablets 5 mg [KYORIN]	KYORIN, Tokyo, Japan	A037	white—slight yellow	uncoated tablet (OD)
#A-6		Amlodipine OD Tablets 5 mg [TOWA]	TOWA, Osaka, Japan	D0278	pale yellow	uncoated tablet (OD)
#A-7		Amlodipine OD Tablets 5 mg [PFIZER]	PFIZER, Tokyo, Japan	EK3802	pale yellow	uncoated tablet (OD)
#A-8		Amlodipine OD Tablets 5 mg [MEIJI]	MEIJI SEIKA, Tokyo Japan	02256	pale yellow	uncoated tablet (OD)
#T-1	Telmisartan	Telmisartan Tablets 20 mg [SAWAI]	SAWAI, Osaka, Japan	17202	white	film-coated tablet
#T-2		Telmisartan Tablets 40 mg [EE]	ELMED EIZAI, Tokyo, Japan	T8RN15	white—slight yellow	film-coated tablet
#T-3		Telmisartan Tablets 40 mg [NIPRO]	NIPRO, Osaka, Japan	18H023	white	film-coated tablet
#T-4		Telmisartan Tablets 40 mg [SAWAI]	SAWAI, Osaka, Japan	17,404	white	film-coated tablet
#T-5		Telmisartan Tablets 80 mg [DSEP]	DAIICHI SANKYO, Tokyo, Japan	889,003	white	film-coated tablet
#T-6		Telmisartan Tablets 80 mg [SAWAI]	SAWAI, Osaka, Japan	118,701	white	film-coated tablet
#T-7		Micardis® Tablets 80 mg	ASTELLAS, Tokyo Japan	889,002	white	film-coated tablet
#T-8		Telmisartan Tablets 20 mg [DSEP]	DAIICHI SANKYO, Tokyo, Japan	889,009	white—slight yellow	uncoated tablet
#T-9		Micardis® Tablets 20 mg	ASTELLAS, Tokyo Japan	889,017	white—slight yellow	uncoated tablet
#T-10		Telmisartan Tablets 40 mg 「JG」	JAPAN GENERIC, Tokyo, Japan	K631K70	white—slight yellow	uncoated tablet
#T-11		Telmisartan Tablets 40 mg [DSEP]	DAIICHI SANKYO, Tokyo, Japan	889,046	white—slight yellow	uncoated tablet
#T-12		Micardis® Tablets 40 mg	ASTELLAS, Tokyo Japan	889,031	white—slight yellow	uncoated tablet
#T-13		Telmisartan OD Tablets 20 mg [SAWAI]	SAWAI, Osaka, Japan	418,702	white—slight yellow	uncoated tablet (OD)
#T-14		Telmisartan OD Tablets 40 mg [SAWAI]	SAWAI, Osaka, Japan	17,315	white—slight yellow	uncoated tablet (OD)
#W-1	Warfarin Potassium	Warfarin Granules 0.2%	EISAI, Tokyo, Japan	22XB54S	dark red	granule
#W-2		Warfarin K Fine Granules 0.2% [NS]	NISSIN, Yamagata, Japan	20021A	white	fine granule

*OD tablets* Orally disintegrating tablets

After storage at room temperature under various conditions, these drugs were used as samples for CL measurement. Packaging included PTP sheets or SDPs, exposed to three light conditions [Condition A, darkness; Condition B, indoor diffused light (approximately 400 lx); and Condition C, exposure to 4,000 lx under standard illuminant D65]. Samples were stored under these conditions ensuring exposed side using printed identification codes on tablets. SDPs were packaged in cellopoly packaging paper, with packaging removed immediately prior to CL measurements. Temperature and humidity without artificial control were monitored with the data logger Ondotori TR-72U (T&D Corporation, Matsumoto, Japan), and they were 20.5—28.0 °C and 39.5—72.6%, respectively.

### CL Measurements

CL Measurements were conducted using a CLA-FS5 with sample chamber CLS-ST4 (Tohoku Electronic Industrial Co., Ltd., Sendai, Japan). Measurements of cumulative light output were taken every minute under nitrogen gas conduction, with a sample chamber temperature of 150 °C, for a maximum of 10 min. Simultaneous luminescence images were captured alongside the CL measurements.

The experiments were repeated twice, yielding similar results; oneof them has been presented here.

## Results

### CL behavior after photooxidation of drugs

**Fig. 1:**
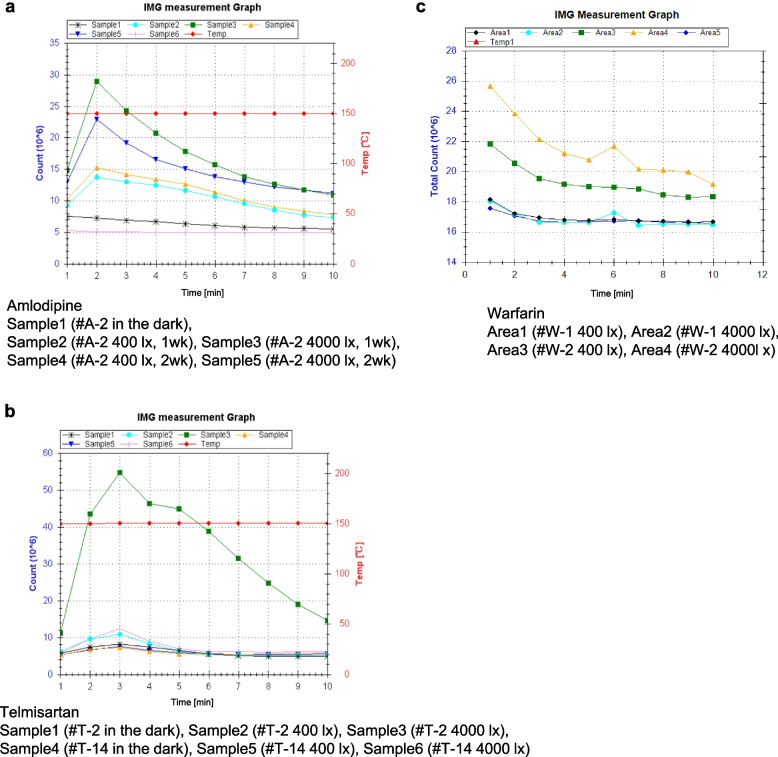
Examples of changes over time in CL during CL measurements. **a** Amlodipine preparations, **b** Telmisartan preparations, **c** Warfarin preparations

The CL of 8 amlodipine preparations, 14 telmisartan preparations, and two warfarin preparations, including brand-name drugs, generic drugs, film-coated tablets, orally disintegrating (OD) tablets, granules, and fine granules, were measured after light exposure. Excluding #A-6, the main CL peak was observed for 1–3 min in the CL profile of each preparation during the 10-min measurement (Fig. 1, Supplementary Figs. S1-S3).

### Detection of changes in the oxidation state on the surface of tablets

**Fig. 2:**
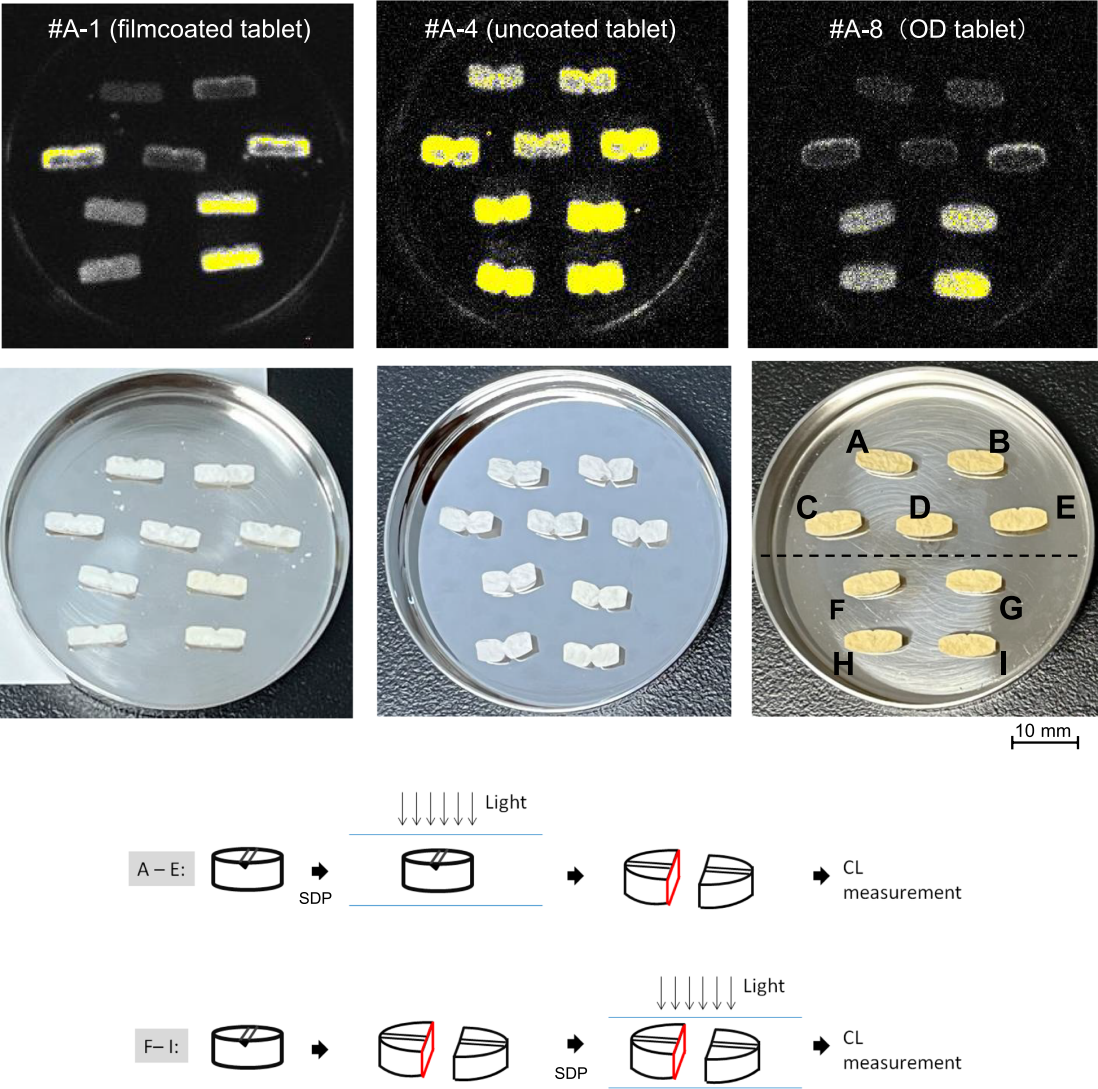
Luminescence images of amlodipine preparation sections. **B–E** After one side of the tablet was exposed to light, the tablet was cut in half for measurement (photoirradiation from top to bottom in the photo). **F–I** Half-tablet sachets (tablets split straight along the score line and stored in single-dose packages). **A** PTP sheet in the dark. **B** Indoor diffused light for 7 days. C: 4000 lx for 7 days. **D** Indoor diffused light for 14 days. E: 4000 lx for 14 days. **F** Indoor diffused light for 7 days. G: 4000 lx for 7 days. **H** Indoor diffused light for 14 days. I: 4000 lx for 14 days

CL was observed on the surface exposed to light, for each amlodipine preparation, whereas luminescence was weak inside the tablets where light did not reach (Fig. 2).

### Detection of differences between tablets

#### Amlodipine tablets

**Fig. 3:**
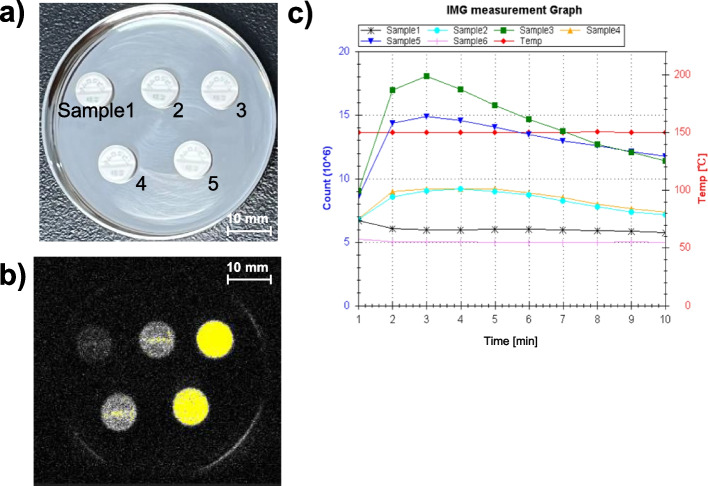
CL of amlodipine preparations. **a** Macroscopic image just before CL measurement, **b** CL image, **c** CL profiles during measurement. Sample 1 (#A-1 PTP), Sample 2 (#A-1 400 lx, one week), Sample 3 (#A-1 4000 lx, one week), Sample 4 (#A-1 400 lx, two weeks), and Sample 5 (#A-1 4000 lx, two weeks)

**Fig. 4:**
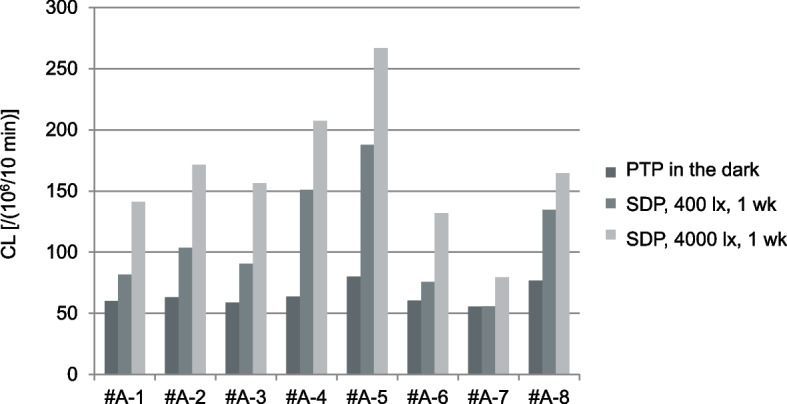
CL of amlodipine preparations. Data are expressed as cumulative CL counts for 10-min measurements

**Fig. 5:**
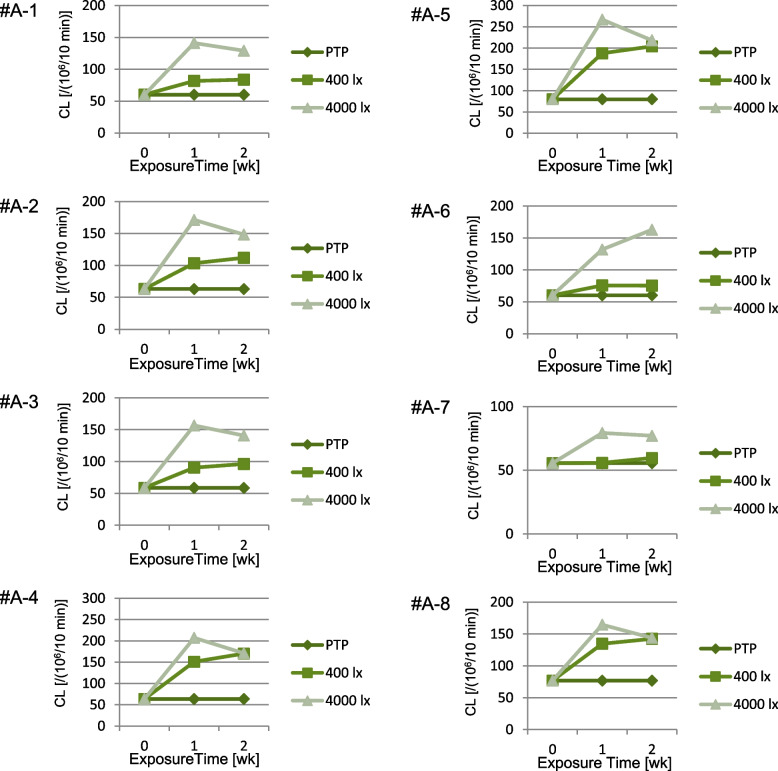
Time course of CL of amlodipine preparations under light exposure. Data are expressed as cumulative CL counts for 10-min measurements

An increase in the CL of each amlodipine preparation was observed with increasing exposure time to light. In the same preparation under identical exposure conditions, CL tended to increase in the order of Condition A < B < C (Figs. 3 and 4, Supplementary Figs. S1, S4, and S5). The CL values in these amlodipine preparations were 55.6–79.9 × 10^6^ [count/10 min] with Condition A, 55.8–187.8 × 10^6^ [count/10 min] with Condition B, and 79.3–266.9 × 10^6^ [count/10 min] with Condition C. Moreover, even when discoloration was not visible to the naked eye, CL increased (Fig. 3). When comparing all amlodipine preparations, differences were observed in the CL increase rate upon exposure, depending on the preparation, and each showed a different reactivity to light. The differences in CL among OD tablets were more pronounced than those among film-coated tablets (Fig. 4). Under Condition C, some samples exhibited lower CL values after 14 d exposure to light compared to those after 7 d (Fig. 5). Samples #A-2 and #A-3 were prepared with the same composition but different brand names and showed similar CL profiles (Supplementary Fig. S1).

#### Telmisartan tablets

**Fig. 6:**
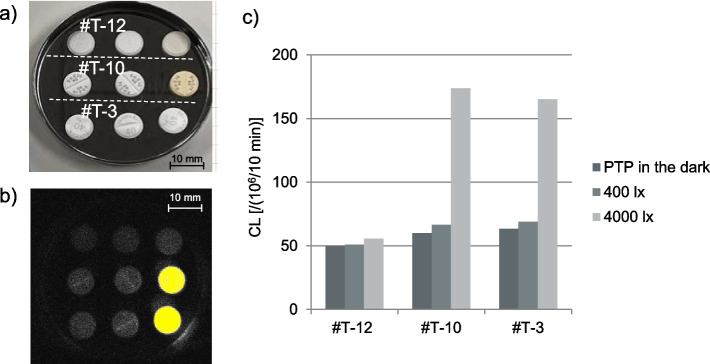
CL of telmisartan preparations. **a** Macroscopic image just before CL measurement, **b** CL image, and **c** Changes in CL of each preparation. Data are expressed as cumulative CL counts for 10-min measurements

Similarly, for the telmisartan preparations, the CL values increased in the order of Condition A < B < C, within the same preparations as shown in Fig. 6 (see details in Supplementary Figs. S2 and S3). The CL values ranged from 46.6–63.3 × 10^6^ [count/10 min] with Condition A, 47.2–68.9 × 10^6^ [count/10 min] with Condition B, and 49.5–329.2 × 10^6^ [count/10 min] with Condition C, indicating an increase in the amount of luminescence increased with light exposure. Differences were observed between preparations in terms of the rate of CL increase with light exposure. During storage under Conditions A–C, minimal brown discoloration was observed in one preparation, #T-10, under Condition C; while, almost no discoloration was visible on the tablets immediately before CL measurement by naked-eye examination. However under Condition C, a significant increase in CL value was observed in #T-3 and #T-10 even though #T-3 showed no discoloration by naked eye (Fig. 6). Variations were noted between preparations in the rate of CL increase across the three light conditions.

**Fig. 7:**
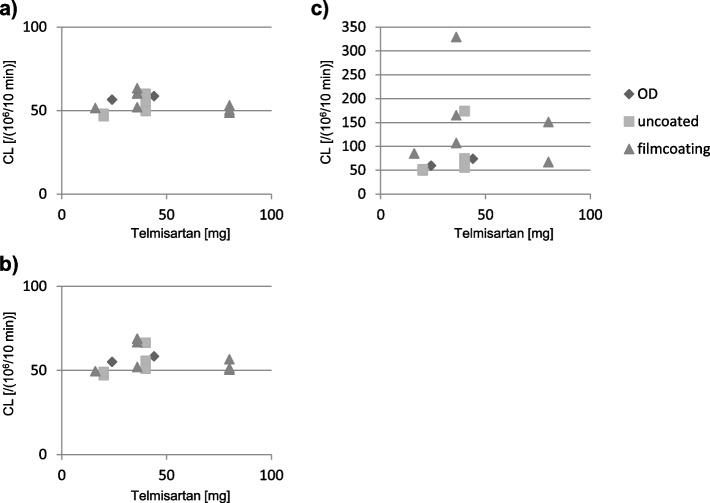
CL values and contents of the active ingredient in telmisartan tablets. **a** PTP in the dark, **b** 400 lx, and **c** 4000 lx for one week. Data are expressed as cumulative CL counts for 10-min measurements

Differences were observed in the CL value increase following light exposure, even among preparations with the same main ingredient and content (Fig. 7). Most film-coated tablet preparations, exhibited a notable increase in CL value under Condition C.

### CL measurements of powdered medicines

**Fig. 8:**
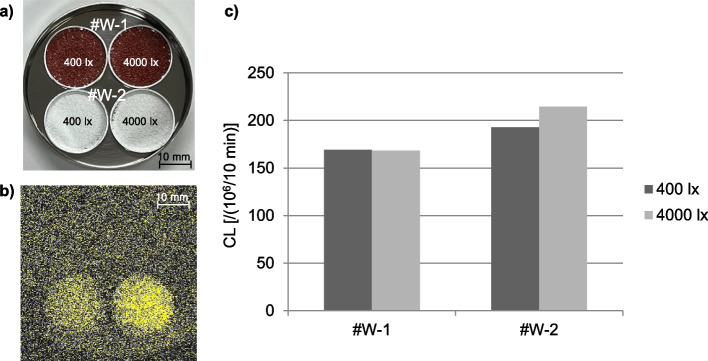
CL of warfarin preparations. **a** Macroscopic image immediately before CL measurement, **b** CL image, **c** Amount of CL after storage

As depicted in Fig. 8, CL can be measured even in powdered preparations. In Sample #W-2, an increase in luminescence was detected after storage under Condition C. Conversely, in #W-1, there was a minimal change in CL observed. And the reproducibility of these results was confirmed.

## Discussion

To the best of our knowledge, this study suggests the first documentation of visual images capturing changes in solid drugs due to oxidation. Image analysis clearly illustrated the progression of oxidation from the tablet surface. Following SDP preparation, variations in CL values were observed depending on storage conditions, demonstrating accelerated photooxidation under harsh light exposure conditions. According to a survey by Mori et al., 64 (68.8%) of 93 target patients required light protection for their regular medications, yet only nine (14.1%) consciously shielded their drugs from light [25]. Figures 2, 3, 6, and 8 underscore the critical importance of light-protective storage for prepared medicines, providing easily comprehensible information for patients.

The samples used in this study were amlodipine, telmisartan, and warfarin, which are widely used in clinical medicine, available in various dosage forms including generics. After exposure to light, distinct CL changes were observed, differing between brand-name drugs and generic drugs or between different generics. Hence, the CL method could be proposed as a novel indicator for drug selection. There is a growing concern that prescription durations will increase owing to rising rates of chronic diseases associated with the aging of populations, highliting the critical need for accessible information on drug stability. Currently, the stability of drugs after SDP preparation is primarily assessed by changes in appearance, such as alternations in color in practical settings. However, there is a clear necessity to develop a more comprehensive system capable of evaluating quality beyond visual indicators such as discoloration. Simple methods, such as CL measurements hold promise for assessing oxidation and ensuring drug stability.

In the context of storage time under Condition C with light exposure, some samples exhibited lower CL at 14 days compared to 7 days (Fig. 5). This suggest that under harsh conditions photooxidation progressed and decomposition continued beyond the initial production of oxidized substances, which are the source of CL generation, reached a plateau.

Increases in CL observed upon exposure to light are believed to be attributable to pharmaceutical additives in each preparation; however, the ingredients were not identified in the present study. Sample #A-6 exhibited a distinct CL profile different from those of the others. It is possible that the presence of a specific pharmaceutical excipient, gum arabic (see Supplementary Table S1), played a role; nevertheless, further investigation is necessary to confirm this hypothesis. Kawabata et al. [26] demonstrated that photooxidation of amlodipine OD tablets led to hydrogen removal from the dihydropyridine ring, forming a pyridine ring. Some of the CL detected in our study may originate from this reaction.

Difference in CL behavior was observed depending on the preparation, including in powdered medicines. Previous reports indicated that light exposure can decrease the warfarin content in fine-grained preparations [27]. In our study, #W-1 exhibited minimal change in CL. The relationship between CL profiles and excipients such as titanium oxide, and red ferric oxide, used in the preparation remains to be clarified.

CL measurements are quick and straightforward. Placing a test sample on a stainless-steel plate inside the sample chamber at a designated temperature, allows measurement of luminous intensity and emission spectrum. These measurements can be conducted over time. If information on the stability of prepared drugs could be swiftly obtained using simple CL measurements, the ripple effects would be enormous. This includes the ability to deliver more stable drug preparations to patients and avoid damaging health or discarding preparations owing to degradation or changes in drug products. Most previous studies on changes in drugs and their properties after preparation focused on single drugs or 2-drug combinations. However, the CL method shows potential for assessing the stability of all combinations involving three or more types of drugs. For instance, Takekuma et al. demonstrated on dissolution tests of rosuvastatin tablets that the dissolution rates of rosuvastatin from tablets stored under normal-humidity with high-temperature condition were significantly lower than those from tablets stored under high-humidity with high-temperature condition, and suggested that the cause was decomposition of rosuvastatin induced by peroxide formation in crospovidone, a pharmaceutical excipient of the tablet, under normal humidity [6]. The CL method could potentially verify such mechanisms. This study focuses solely on the effects of light during storage. It is essential to further investigate the CL method under different storage conditions, including temperature and humidity variations, which also influence the stability of preparations.

For liquid preparations, the volatility effects needs to be considered for measurements in the high-temperature sample chamber of the CL-measuring device, and quenching occurs from water. Therefore, applying the CL method under current measurement conditions is considered difficult. Liquid medicine and ointments were not included in the present study, necessitating further investigations. Additionally, it’s important to note that CL measurements are destructive tests and provide values for the entire preparation rather than focusing solely on active ingredients. Caution is required when performing CL measurements.

The CL method offers the advantage of visually confirming susceptibility to oxidation through image analysis, which is a significant feature of this technique. It enables multiple simultaneous analyses of CL from tablets and capsules of various sizes and shapes, promising the development of new assessment methods. Moreover, the method exhibits high sensitivity; allowing for early detection of photooxidation, and potentially reducing the time required for photostability tests [28, 29]. Furthermore, also it holds promise for developing sustainable testing approaches that consider cost reduction, environmental impact and energy conservation.

Based on the findings of the present study, the CL method may contribute to addressing the challenge of insufficient drug stability information despite its research limitations.

Because the CL method evaluates the stability of the entire preparation (mixed system), its results may not correlate with data from conventional methods. Detailed experiments are necessary to ascertain which components contribute to the CL values and to what extent for each preparation. Further investigation should include stability after tablets are ground, assessing the stability of powdered medicine mixtures, and exploring various conditions, such as the impact of pharmaceutical additives, are warranted.

## Conclusions

The visual capture of tablet surface oxidation due to light exposure was achieved for the first time using the CL method. CL values attributed to photooxidation increased upon light exposure of the tablets after SDP preparation. Furthermore, after SDP preparation, differences were observed in the rate of increase in the CL values after light exposure, depending on the pharmaceutical brand and susceptibility to oxidation. Some preparations showed increased luminescence even without visible discoloration, suggesting that drug degradation from photooxidation can be sensitively assessed using CL measurements. Further research is warranted to establish the CL method as a reliable tool for assessing the stability of preparations in clinical settings.

### Supplementary Information

Supplementary Material 1: Supplementary Table S1. The pharmaceutical excipients listed in the package inserts. Supplementary Fig. S1. CL profiles of amlodipine tablets. Sample 1 (PTP), Sample 2 (400 lx for one week), Sample 3 (4000 lx for one week), Sample 4 (400 lx for two weeks), and Sample 5 (4000 lx for two weeks). Supplementary Fig. S2. CL profiles of film-coated telmisartan tablets. Supplementary Fig. S3. CL profiles of uncoated telmisartan tablets. Supplementary Fig. S4. Photographs of amlodipine tablets immediately before CL measurements. Supplementary Fig. S5. CL images of amlodipine tablets.

## Data Availability

All data generated or analyzed in this study have been included in the published article.

## Abbreviations

CL: Chemiluminescence
CLA: Chemiluminescence analyzer
OD: Orally disintegrating
PTP: Press-through package
SDP: Single-dose package
