# Supplementary material for: Chemiluminescence method for evaluating photooxidative degradation of dispensed drugs: a potential new drug information tool
Source: J Pharm Health Care Sci. 2024 Jul 24;10:44. doi: 10.1186/s40780-024-00365-7 (PMC11267834; doi:10.1186/s40780-024-00365-7)
Supplement: Supplementary file 1 — Supplementary Material 1: Supplementary Table S1. The pharmaceutical excipients listed in the package inserts. Supplementary Fig. S1. CL profiles of amlodipine tablets. Sample 1 (PTP), Sample 2 (400 lx for one week), Sample 3 (4000 lx for one week), Sample 4 (400 lx for two weeks), and Sample 5 (4000 lx for two weeks). Supplementary Fig. S2. CL profiles of film-coated telmisartan tablets. Supplementary Fig. S3. CL profiles of uncoated telmisartan tablets. Supplementary Fig. S4. Photographs of amlodipine tablets immediately before CL measurements. Supplementary Fig. S5. CL images of amlodipine tablets. [file 40780_2024_365_MOESM1_ESM.pdf]

Table S1. The pharmaceutical excipients listed in the package inserts.

	#A-								#T-														#W-	
	1	2	3	4	5	6	7	8	1	2	3	4	5	6	7	8	9	10	11	12	13	14	1	2
Diameter [mm]	8.1	8.1	8.0	5.9<br>8.7	7.5	8.5	7.0	7.0	6.1	8.1	8.1	8.1	10.0	10.1	10.0	6.0	6.0	8.0	8.0	8.0	8.0	10.0	-	-
Aminoalkyl Methacrylate Copolymer E							●														●	●		
Anhydrous Dibasic Calcium Phosphate	●	●	●	●														●						
Aspartame					●	●		●																
Calcium Silicate																		●						
Carnauba Wax	●	●	●						●	●	●	●		●										
Corn Starch											●										●	●		
Croscarmellose sodium								●										●						
Crospovidone					●	●	●	●													●	●		
Dextrin					●	●																		
Dimethylpolysiloxane, Silicon Dioxide Mixture							●																	
D-Mannitol					●	●	●	●	●	●	●	●	●	●	●			●			●	●	●	
Erythritol																●	●		●	●				
Flavor					●	●	●																	
Gum Arabic						●																		
Hydrated Silicon Dioxide							●																	
Hydroxypropyl Starch								●																
Hydroxypropylcellulose					●			●		●													●	●
Hypromellose	●	●	●					●	●	●	●	●	●	●	●						●	●	●	
Lactose Hydrate								●															●	●
Lauromacrogol									●			●		●							●	●		
Light Anhydrous Silicic Acid						●		●	●	●	●	●	●	●	●	●	●		●	●	●	●	●	
l-Menthol						●		●																
Macrogol 6000									●	●	●	●	●	●	●						●	●		
Magnesium Aluminometasilicate																					●	●		
Magnesium Stearate	●	●	●	●	●	●	●	●	●	●	●	●	●	●	●	●	●	●	●	●	●	●		●
Meglumine									●	●	●	●	●	●	●	●	●		●	●	●	●		
Methacrylic Acid Copolymer L								●																
Microcrystalline Cellulose	●	●	●	●	●		●	●																
New Coccine								●																
Polyoxyethylene(160)Polyoxypropylene(30) Glycol													●		●	●	●		●	●				
Polyoxyl 40 Stearate										●														
Polysorbate 80											●													
Povidone																		●						
Powder Hydrogenated Maltose Starch Syrup								●																
Red Ferric Oxide							●																●	
Sodium Hydroxide											●							●						
Sodium Lauryl Sulfate							●																	
Sodium Starch Glycolate	●	●	●	●																				
Stearic Acid							●																	
Sucralose							●														●	●		
Synthetic Aluminum Silicate								●																
Talc	●	●	●			●	●		●		●	●	●	●	●								●	
Tartrazine								●																
Tetrasodium Edetate Dihydrate								●																
Thaumatin					●			●																
Titanium Oxide	●	●	●						●	●	●	●	●	●	●								●	

#A-1

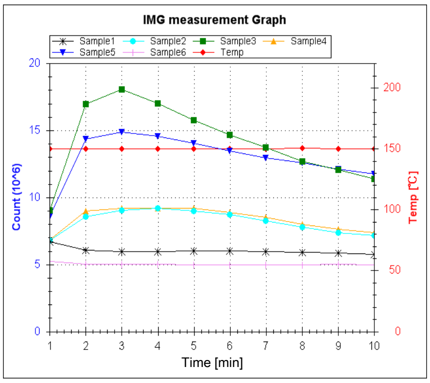

#A-5

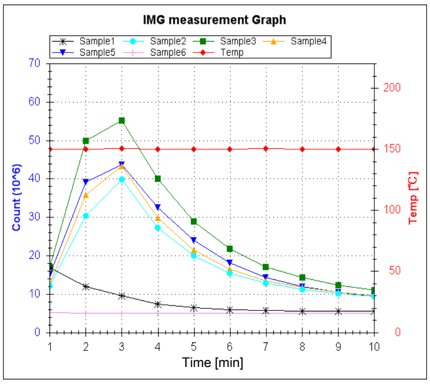

#A-2

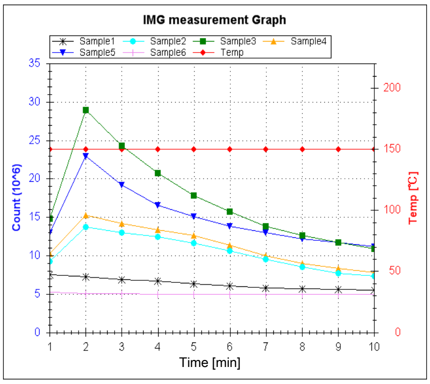

#A-6

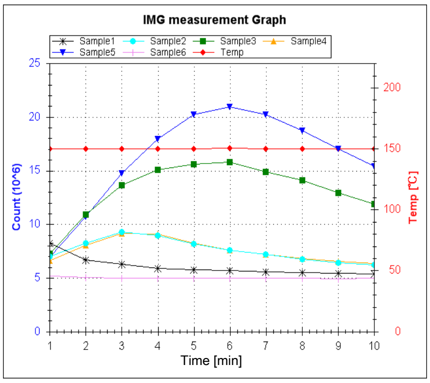

#A-3

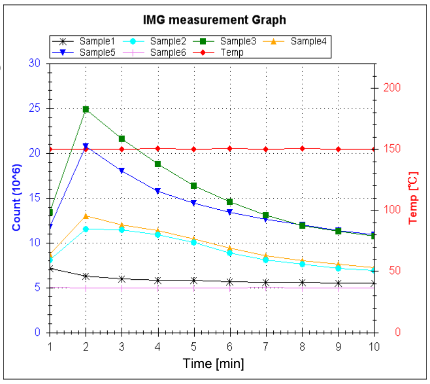

#A-7

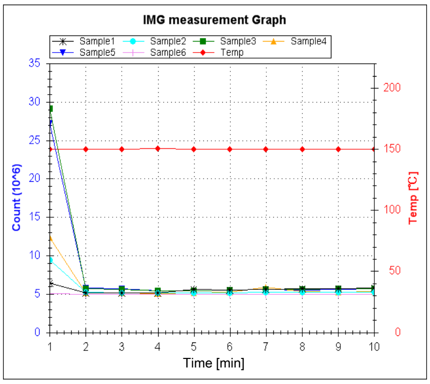

#A-4

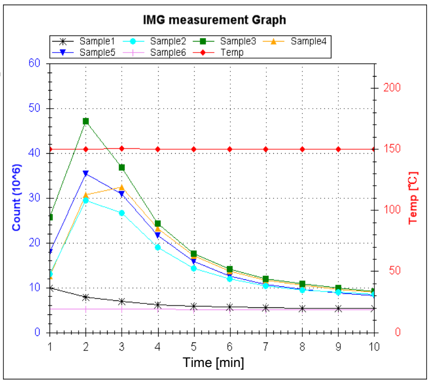

#A-8

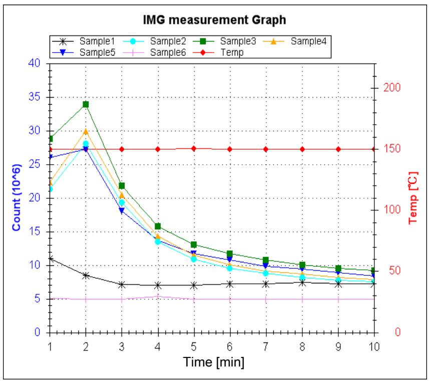

Fig. S1. CL Profiles of Amlodipine Tablets

Sample1 (PTP), Sample2 (400 lx for one week), Sample3 (4000 lx for one week), Sample4 (400 lx for two weeks), and Sample5 (4000 lx for two weeks).

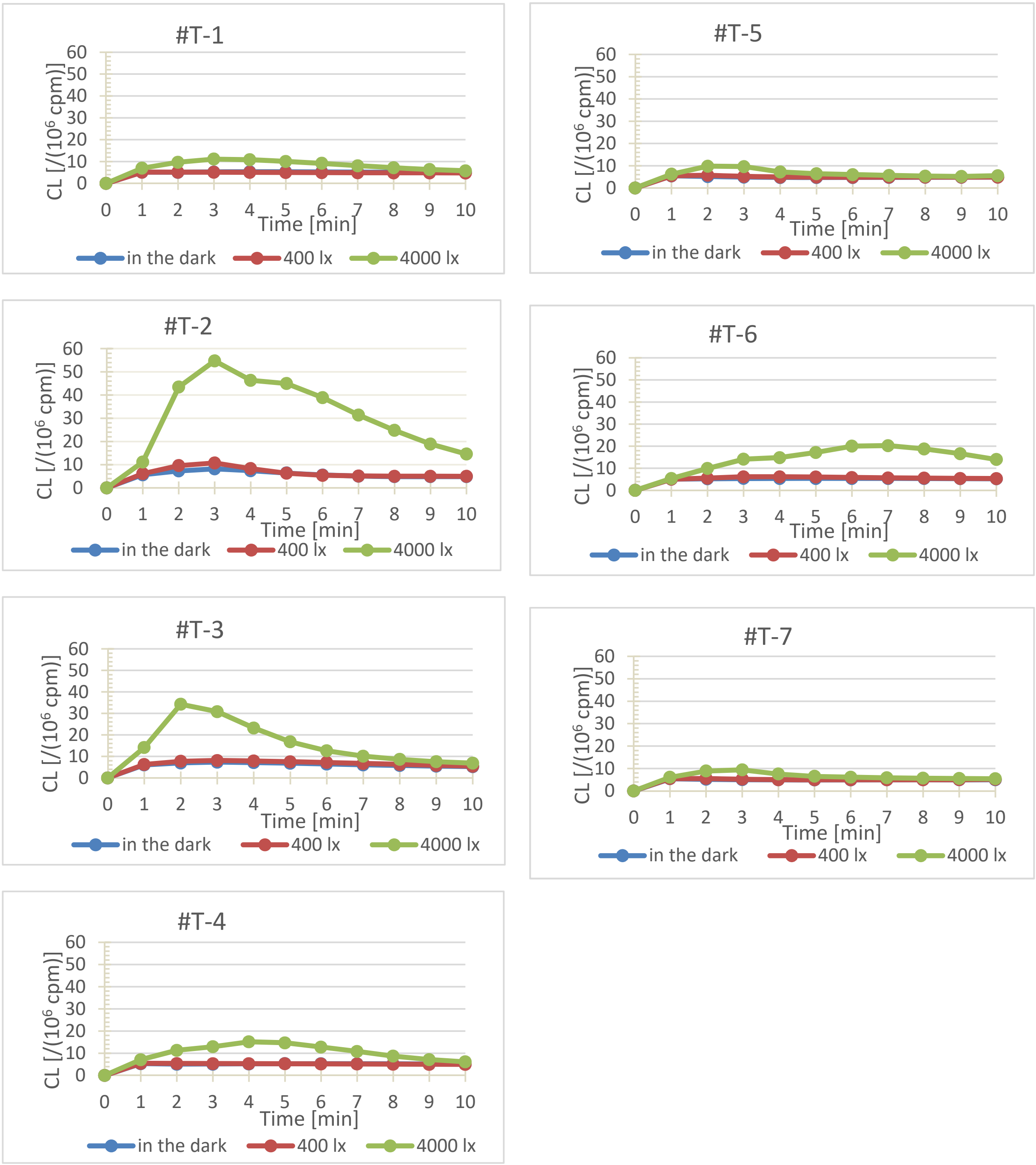

Fig. S2. CL Profiles of Film-Coated Telmisasrtan Tablets

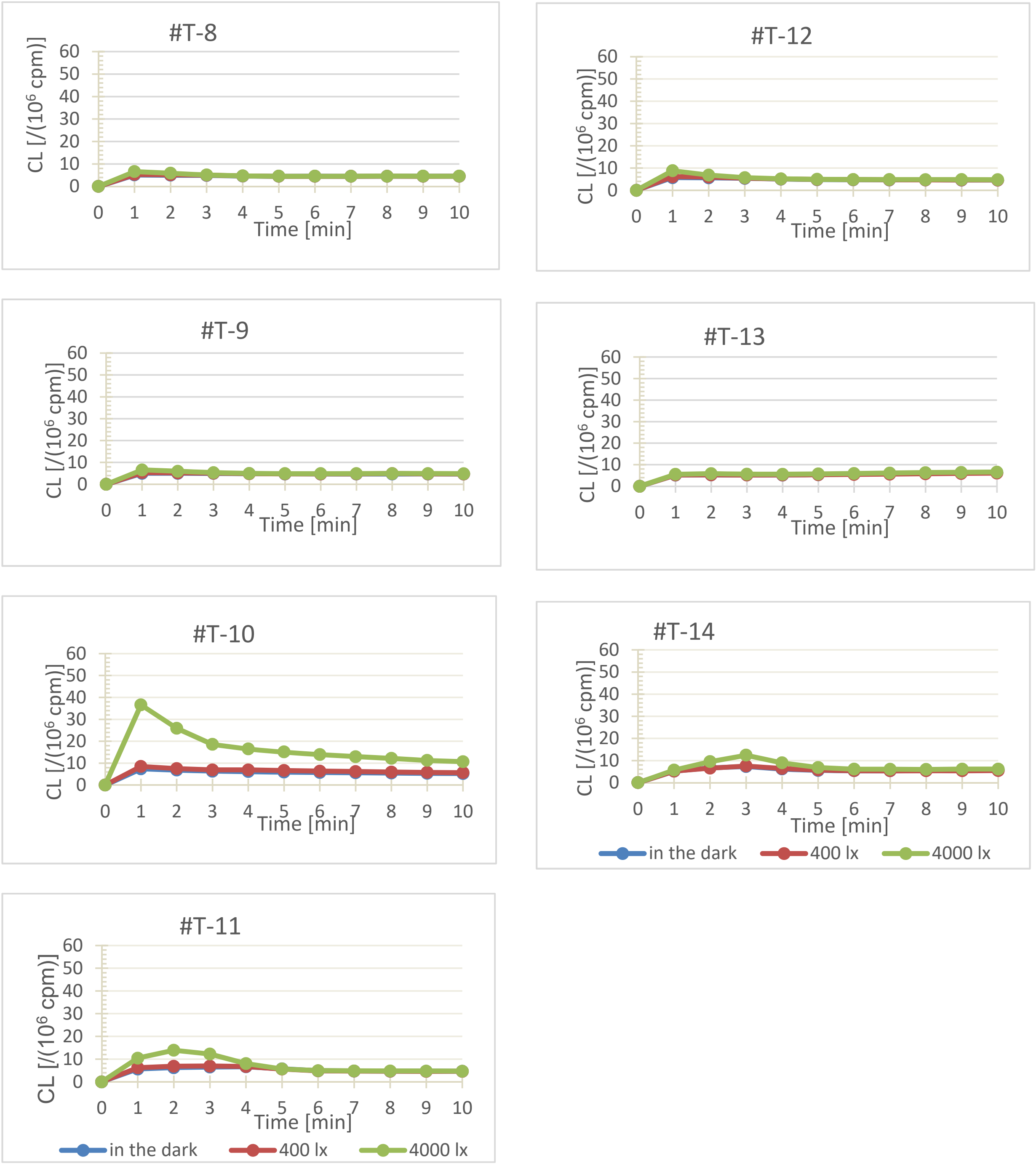

Fig. S3. CL Profiles of Uncoated Telmisasrtan Tablets

#A-1 #A-5

#A-2 #A-6

#A-3 #A-7

#A-4 #A-8

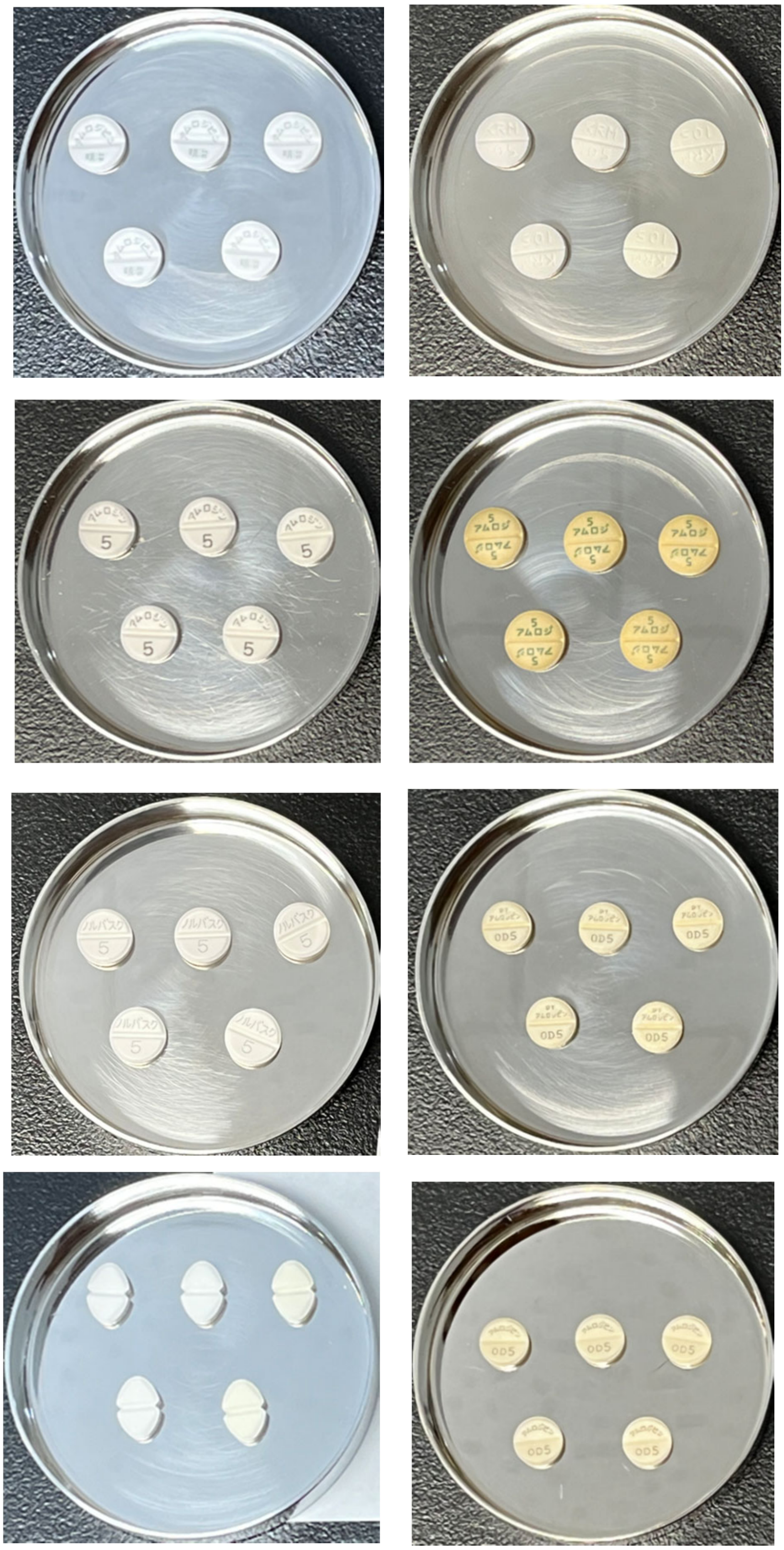

Fig. S4. Photographs of Amlodipine Tablets Immediately before CL Measurements

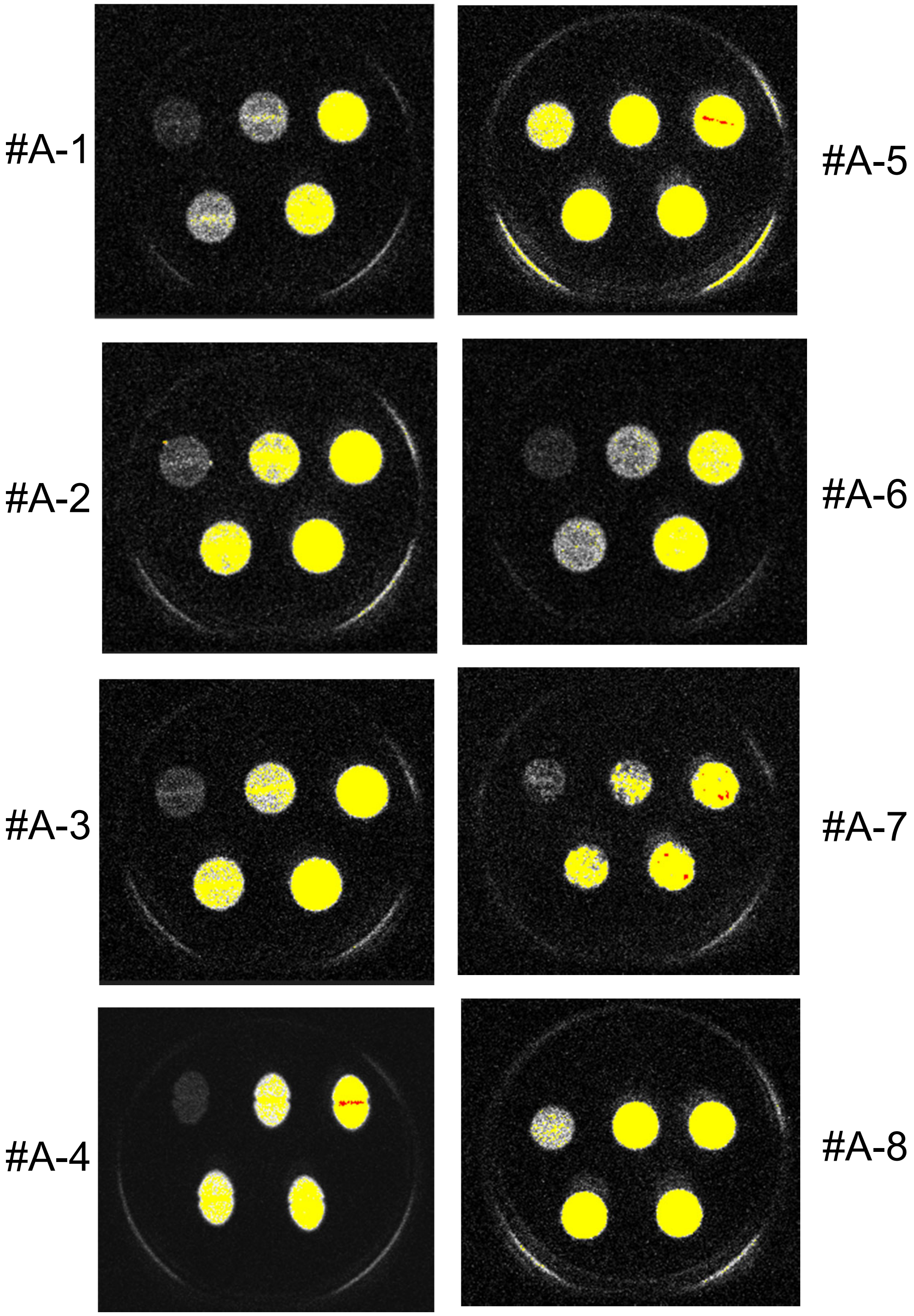

Fig. S5. CL Images of Amlodipine Tablets
